# Supplementary material for: Dissipative soliton generation and real-time dynamics in microresonator-filtered fiber lasers
Source: Light Sci Appl. 2022 Oct 12;11:296. doi: 10.1038/s41377-022-00998-z (PMC9556569; doi:10.1038/s41377-022-00998-z)
Supplement: Supplementary file 1 — Supplementary Information for “Dissipative soliton generation and real-time dynamics in microresonator-filtered fiber lasers” [file 41377_2022_998_MOESM1_ESM.docx]

Supplementary Information for “Dissipative soliton generation and real-time dynamics in microresonator-filtered fiber lasers”

Mingming Nie^1, #, *^, Bowen Li^1, #^, Kunpeng Jia^2^, Yijun Xie^1^, Jingjie Yan^1^, Shining Zhu^2^, Zhenda Xie^2^, and Shu-Wei Huang^1, *^

^1^Department of Electrical, Computer and Energy Engineering, University of Colorado Boulder, Boulder, Colorado 80309, USA

^2^School of Electronic Science and Engineering, National Laboratory of Solid State Microstructures, College of Engineering and Applied Sciences, School of Physics, and Collaborative Innovation Center of Advanced Microstructures, Nanjing University, Nanjing 210093, China

^#^These authors contribute equally

^*^Corresponding author: [mingming.nie@colorado.edu](mailto:mingming.nie@colorado.edu), [shuwei.huang@colorado.edu](mailto:shuwei.huang@colorado.edu)

This Supplementary Information to “Dissipative soliton generation and real-time dynamics in microresonator-filtered fiber lasers” provides additional information for the main text.

- In Section S1, we provide the theory to model the microresonator-filtered fiber lasers.
- In Section S2, we provide the bifurcation of the normalized LLE to show the transmission curves.
- In Section S3, we study the influence of microresonator GVD on the soliton bandwidth.
- In Section S4, we study the dispersive wave emission caused by the different GVD signs of the microresonator and the EDFA.
- In Section S5, we study the influence of intracavity spectral filtering on soliton.
- In Section S6, we phenomenologically explain the soliton self-starting and self-stabilization.
- In Section S7, we provide spectra and temporal traces for other comb states.
- In Section S8, we provide the details for the time magnifier setup.
- In Section S9, we explain the phase shift at edges of the bandpass filter.
- In Section S10, we simulate XPM induced combs to match the experimental results.
- In Section S11, we discuss the long-term stability of the soliton microcombs.
- In Section S12, we provide the temporal evolution from the time magnifier system for state IV in Fig. 4a.
- In Section S13, we provide the details for linewidth measurement of the FFP microresonator.

**S1: Theory and model**

The nonlinearity can be neglected in the EDFA due to the high repetition rate and low pulse energy. Therefore, pulse output from the microresonator drop port experiences only gain and group velocity dispersion (GVD) in the EDFA. Assuming the fiber length (*mL*) of the EDFA is multiple of the length of the microresonator (*L*), the relationship between the optical field in the EDFA (*B*) satisfies

$\widehat{B}\left(mL,\omega\right)=\widehat{B}\left(0,\omega\right)\exp\left\{\left[-\alpha_{1}+\left(1-\frac{\omega^{2}}{\Omega_{\text{g}}^{2}}\right)g+i\frac{\beta_{2}^{'}}{2}\omega^{2}\right]mL\right\}$ (S1)

$g=\frac{g_{0}}{1+\int_{-t_{\text{R}}/2}^{t_{\text{R}}/2} \left|B\right|^{2}d\tau/E_{\text{sat}}}$ (S2)

where $\widehat{B}$ is the Fourier transform of field *B*, *α*_1_ is the propagation loss in the EDFA, *g* is the power-dependent gain, *ω* is the angular optical frequency, *Ω_g_* is the gain bandwidth, $\beta_{2}^{'}$ is the net GVD of the EDFA, *g*_0_ is the small-signal gain, *E*_sat_ is the saturated energy, *t*_R_ is the cavity roundtrip time. To form a laser cavity, the output from the microresonator (*A*) propagates in the EDFA and the amplified field *B* serves as the pump of the microresonator *A*_p_, which can be expressed as

(S3)

(S4)

where represents the Fourier transform.

The intracavity field in the microresonator can still be modeled using Lugiato-Lefever equation (LLE)

$t_{\text{R}}\frac{\partial A\left(t,\tau\right)}{\partial t}=\left[-\left(\alpha+i\delta_{0}+i\frac{\beta_{2}L}{2}\frac{\partial^{2}}{\partial\tau^{2}}\right)+i\gamma L\left|A\right|^{2}+r\cdot2i\gamma LP_{\text{avg}}\right]A+\sqrt{\theta}A_{\text{p}}$ (S5)

where parameter *r* determines whether the microresonator is with Fabry–Pérot (FP) configuration (*r*=1) or not (*r*=0), *P*_avg_ is the average intracavity intensity.

**S2: Bifurcation of the normalized LLE**

The bifurcation in Fig. 1c and 1d in the main text is achieved by solving the normalized LLE and conducting linear stability analysis. The normalized LLE for ring microresonator is given by [1]

$\frac{\partial A\left(t,\tau\right)}{\partial t}=\left[-\left(1+i\text{Δ}+i\eta\frac{\partial^{2}}{\partial\tau^{2}}\right)+i\left|A\right|^{2}\right]A+S$ (S6)

where *η*=-1 represents anomalous dispersion while *η*=1 represents normal dispersion. The input power *X* is defined as *X*=|*S*|^2^ while the intracavity power *Y* is defined as *Y*=|*A*|^2^.

With a fixed input power of *X*=30, we plot the bifurcation and corresponding linear stability of the homogeneous solution and soliton solution [2]. This analysis can also be applied to the FP microresonators. As shown in Fig. S1a for anomalous-GVD microrings (*η*=-1), at red detuning, the microring favors stable soliton with high peak power thus high transmission, instead of the stable homogeneous solution (CW) with much lower power (lower branch with blue line). With scanned detuning from the blue side the red side, the output will experience CW, modulation instability and eventually soliton state. As for normal-GVD microresonators (*η*=1), it is infeasible to access modulation instability (Fig. S1b) without the help of perturbation such as avoided mode crossings [3].


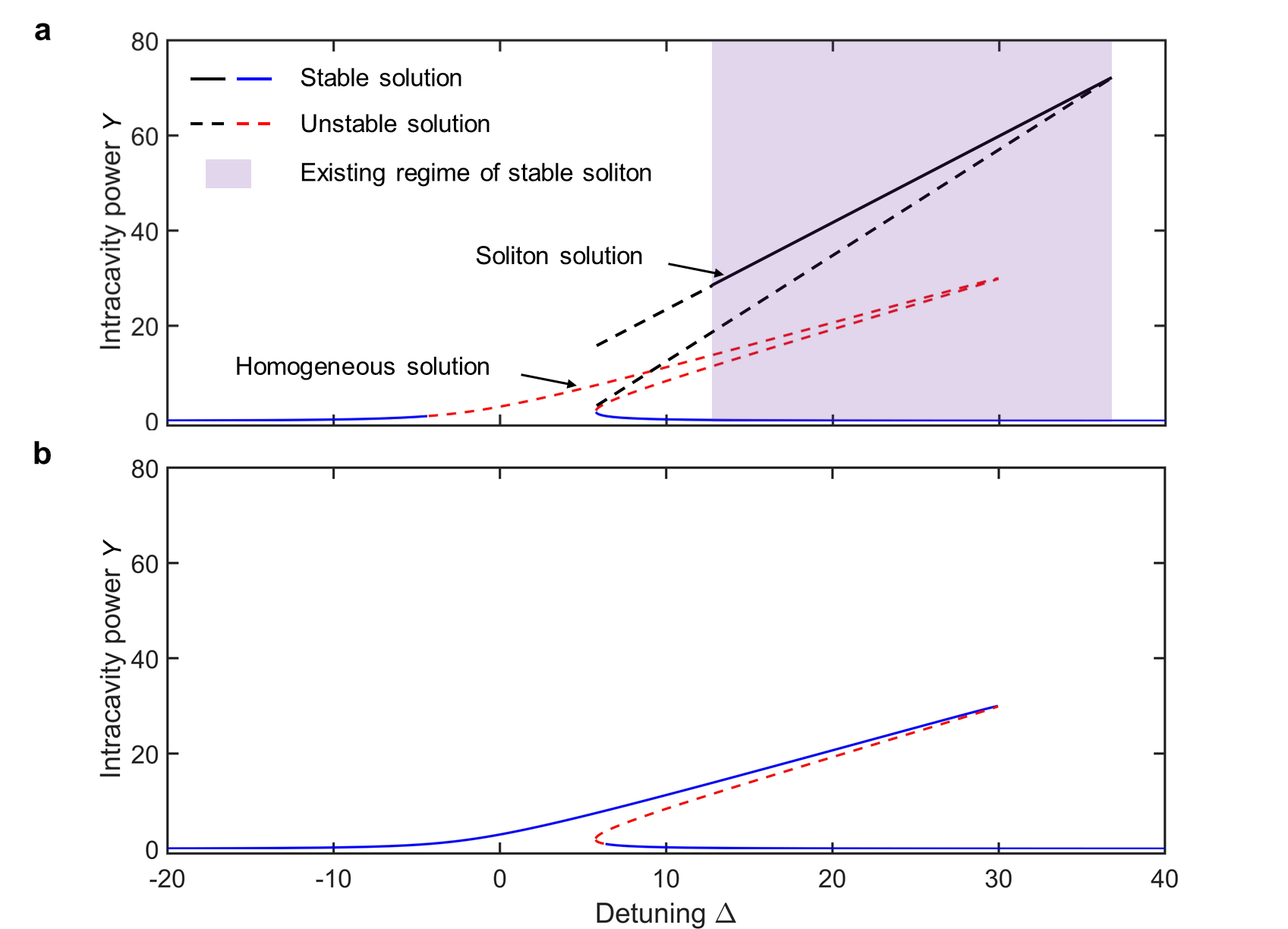


Fig. S1. Bifurcation and linear stability analysis of the homogeneous solutions and soliton solution for microrings with anomalous GVD (a) and normal GVD (b).

**S3: Influence of microresonator GVD on the soliton bandwidth**

Figure S2 shows the influence of microresonator’s GVD on the soliton bandwidth when the EDFA’s GVD is set to be zero. Microresonators with small GVD in microresonator-filtered fibers can lead to large soliton bandwidth.


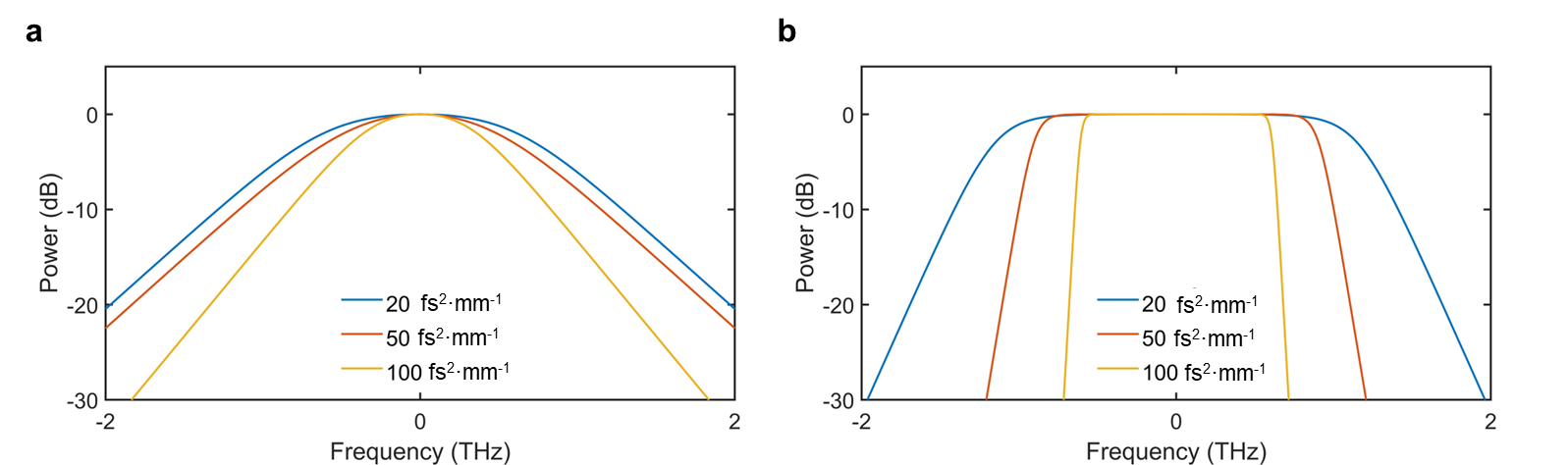


Fig. S2. Influence on the soliton bandwidth from the GVD of the microresonator. (a) Microresonators with different anomalous GVDs; (b) Microresonators with different normal GVDs. The GVD of the EDFA is assumed to be zero for simplicity.

**S4: Dispersive wave emission**

Opposite signs of both GVDs (microresonator GVD and EDFA GVD) will lead to dispersive wave emission, causing soliton perturbation and destabilization, as shown in Fig. S3. For anomalous-GVD microresonators in Fig. 1e, stable soliton can not exist with normal EDFA GVD >40 fs^2^·mm^-1^ (Fig. 1e), while for normal-GVD microresonators in Fig. 1f, soliton can not keep stable with large anomalous EDFA GVD >-100 fs^2^·mm^-1^.


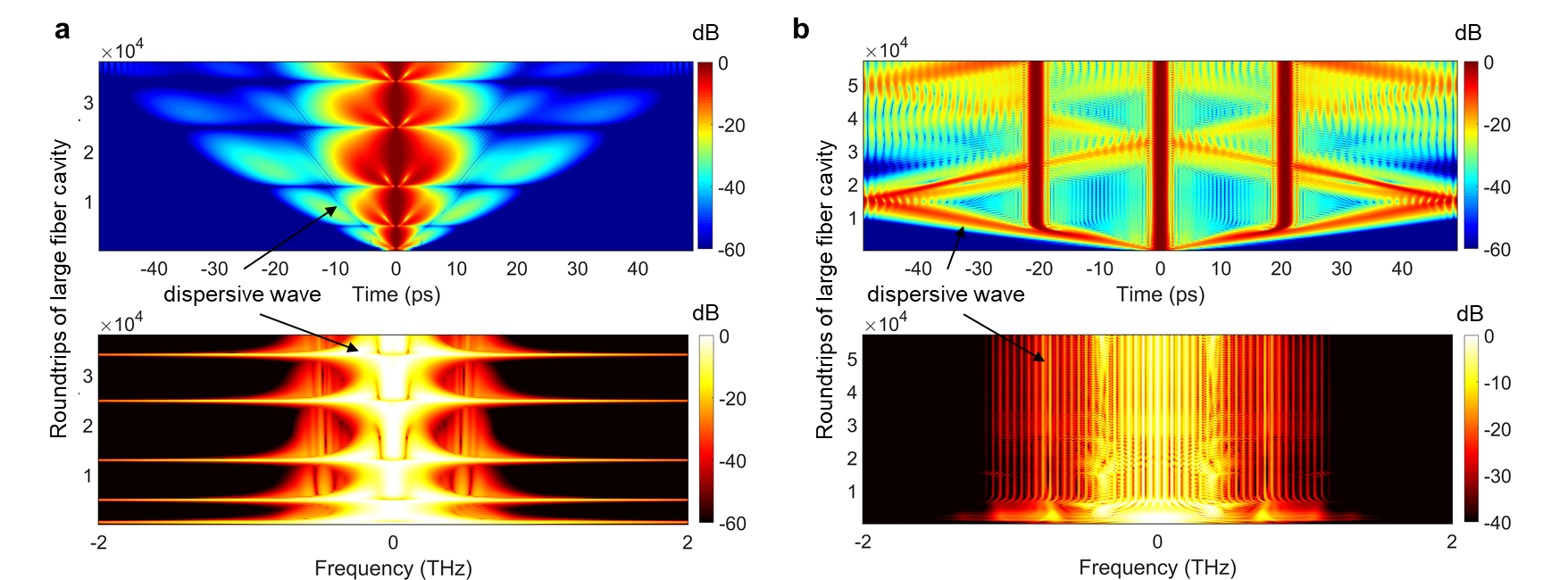


Fig. S3. Dispersive wave emission. (a) anomalous-GVD microresonator nested in normal-GVD fiber cavity; (b) normal-GVD microresonator nested in anomalous-GVD fiber cavity. Top: temporal evolution; bottom: spectral evolution.

**S5: Influence of intracavity spectral filtering**


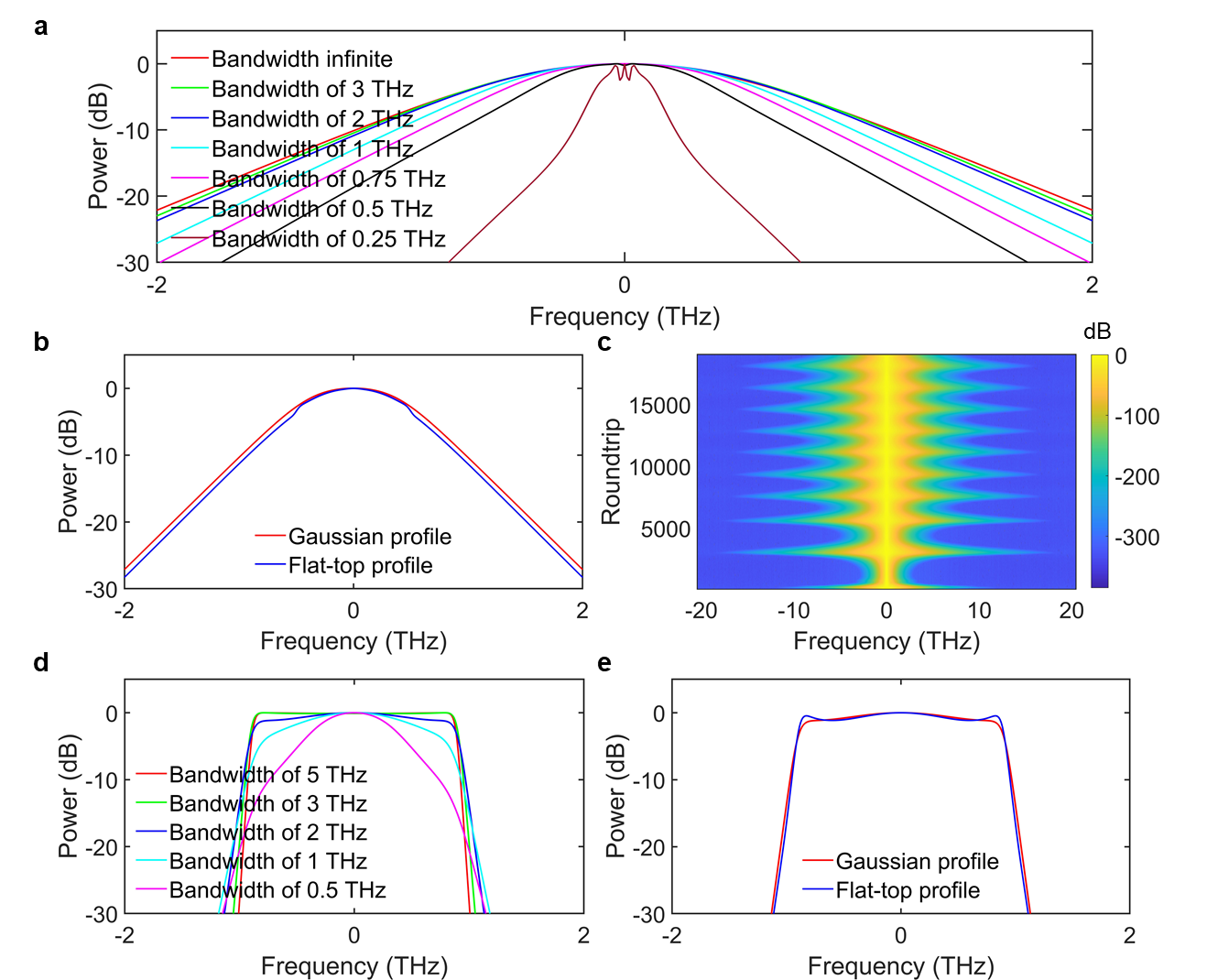


Fig. S4. The effect of intracavity spectral filter with different bandwidth on soliton when the GVD of large fiber cavity equals zero. (a)-(c) Microresonators with anomalous GVD. (a) soliton bandwidth with Gaussian bandpass filter and different filter (3-dB) bandwidth. (b) comparison of soliton spectra with different filter spectral profiles. (c) breathing soliton. (d)(e) Microresonators with anomalous GVD. (d) soliton bandwidth with Gaussian bandpass filter and different filter (3-dB) bandwidth. (e) comparison of soliton spectra with different filter spectral profiles.

Compromise should be made between the stable soliton bandwidth and intracavity spectral filtering since too narrow bandwidth will in turn lift the pump threshold, lower the soliton mode efficiency, narrow the soliton bandwidth and even prevent the soliton generation.

The intracavity BPF will affect both the soliton bandwidth and soliton stability. The effect of intracavity spectral filter with different bandwidth on the soliton is shown in Fig. S4. Zero GVD for the EDFA is assumed here for simplicity. For microresonators with anomalous GVD, the solitons result from the balance between nonlinearity and GVD and corresponding soliton bandwidth is dominated by the two factors and not by the intracavity filter. As shown in Fig. S4a, when the filter is broad enough, it will not change the soliton bandwidth too much, especially for the 3-dB bandwidth. In addition, different filter spectral profiles with the same 3-dB bandwidth will not influence the final soliton bandwidth, as shown in Fig. S4b. Besides, solitons with too narrowband filter will experience breathing behavior (Fig. S3c) and even complete cease. As for microresonators with normal GVD, when the filter is broad enough, the dissipative soliton bandwidth is eventually limited by the gain bandwidth (Fig. S4d). Furthermore, spectral filter slightly affects the soliton spectrum especially the spectral edge (Fig. S4e).

**S6: Self-starting and self-stabilization behavior**

According to our experimental results and the preprint paper [4], in the microresonator-filtered laser cavities, the employment of active gain is the key to the self-starting and self-stabilized behavior. In general, the self-starting dynamics and self-stabilization are determined by the lasing frequency shift and detuning change due to both the thermal nonlinearity in the microresonator and the optical path length change in the gain fiber.

There are two main contributions in the optical-path-length change for the gain fiber: (i) the resonant part determined by the saturation effect in the fiber and (ii) the thermal part stemming from the fiber heating due to the excited-state absorption and Stokes losses [5]. According to the experimental result in Fig. S5, the optical-path-length change of the gain fiber is ~50 µm when the EDFA is on with 980-nm pump power of 0.8 W, which is mainly due to the fiber heating.

The general guideline to the self-starting behavior is to engineer the global parameters to strike a balance between the frequency shifts induced by both the thermal nonlinearity in the microresonator and the optical-path-length change in the gain fiber, ensuring the final stable detuning is at the soliton existing red-detuned regime. The global parameters include the gain (determined by the pump power), gain saturation (determined by the signal power and the pump power), the cavity loss and the initial detunings (determined by the delay line). If the global parameters are not correct, the system will enter into multiple possible states, such as continuous-wave state, Turing pattern and chaotic state.

In the experiment, we first operate the system at a relatively high EDFA pump power and adjust the delay line to find a soliton state. If the delay line is at the correct position, soliton can emerge during the process of adjusting the delay line. However, soliton might not self-start by just turning on the EDFA. Therefore, we have to carefully and fine adjust the EDFA pump power, the coupling ratio and the delay line around this operating point.


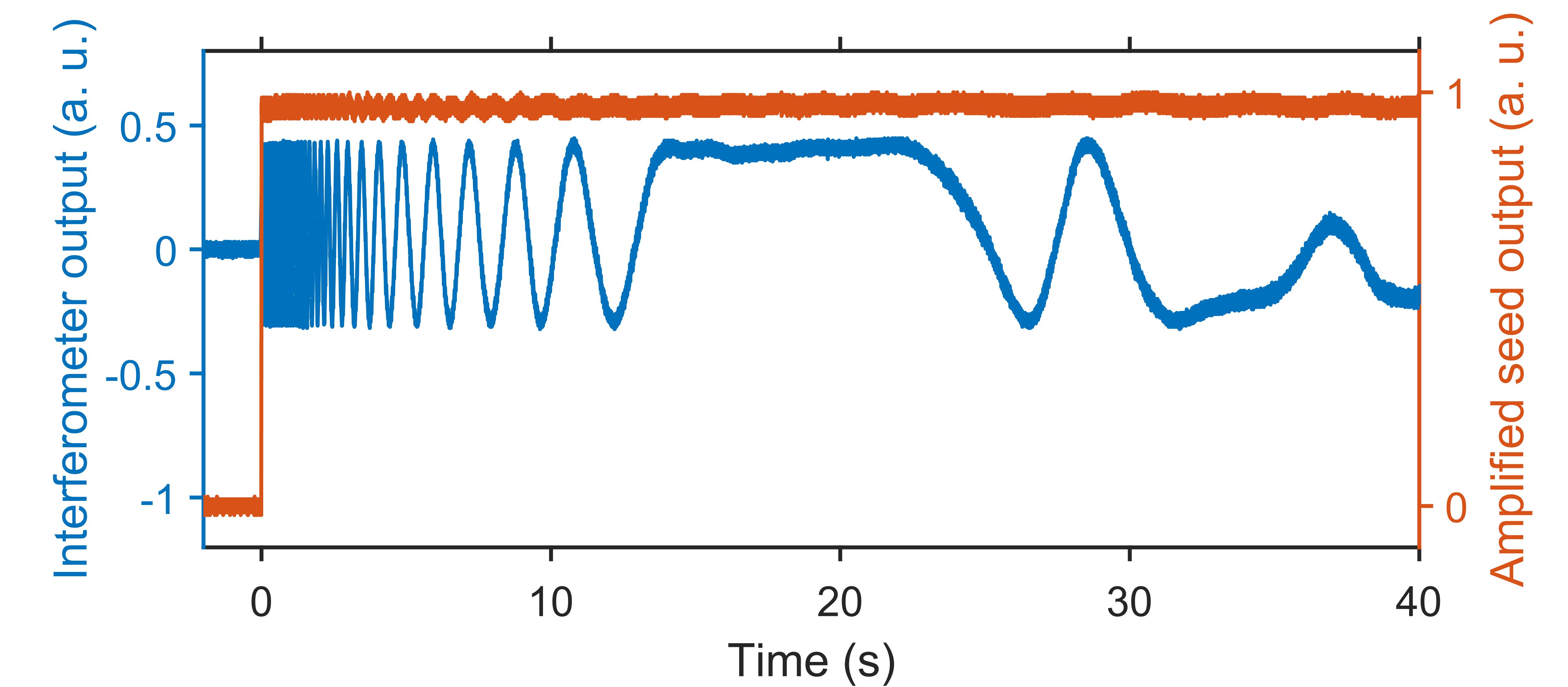


Fig. S5. An interferometer is utilized to measure the optical path length change when the EDFA is on. One of the arms is made of the EDFA, while the other arm is made of passive single-mode fiber with the same fiber length. The seed power of the EDFA is 2 mW, similar to the intracavity power before entering into the in-loop EDFA.

The self-stabilization is achieved by self-fixing the detunings at the same position under external perturbations. This is possible when the lasing frequencies of the large fiber cavity and the microresonator resonance shift towards the same direction with the same order of magnitude, which results from the optical-path-length change by the gain saturation in the gain fiber and the thermal nonlinearity in the microresonator, respectively. For example, a sudden power increase will lead to the stronger gain saturation effect and the optical-path-length increase in the gain fiber. Then the lasing frequencies of the fiber cavity will go towards the longer wavelength. At the same time, this frequency shift will in turn increase the soliton energy, heat the microresonator and shift the resonance also to the longer wavelength. If the global parameters are correct, for example, the gain is correct, the optical-path-length change induced frequency shift can have the same amount compared to the microresonator resonance shift. Therefore, the detunings can be fixed at almost the same red detuned value and stabilize the soliton.

**S7: Spectra and temporal traces for other comb states**

Spectra and calibrated temporal traces for other comb states are shown in Figs. S6 and S7, respectively. The residual intensity variation on the calibrated single-shot waveform is attributed to the finite signal-to-noise ratio of time-lens output waveform as well as the response function.


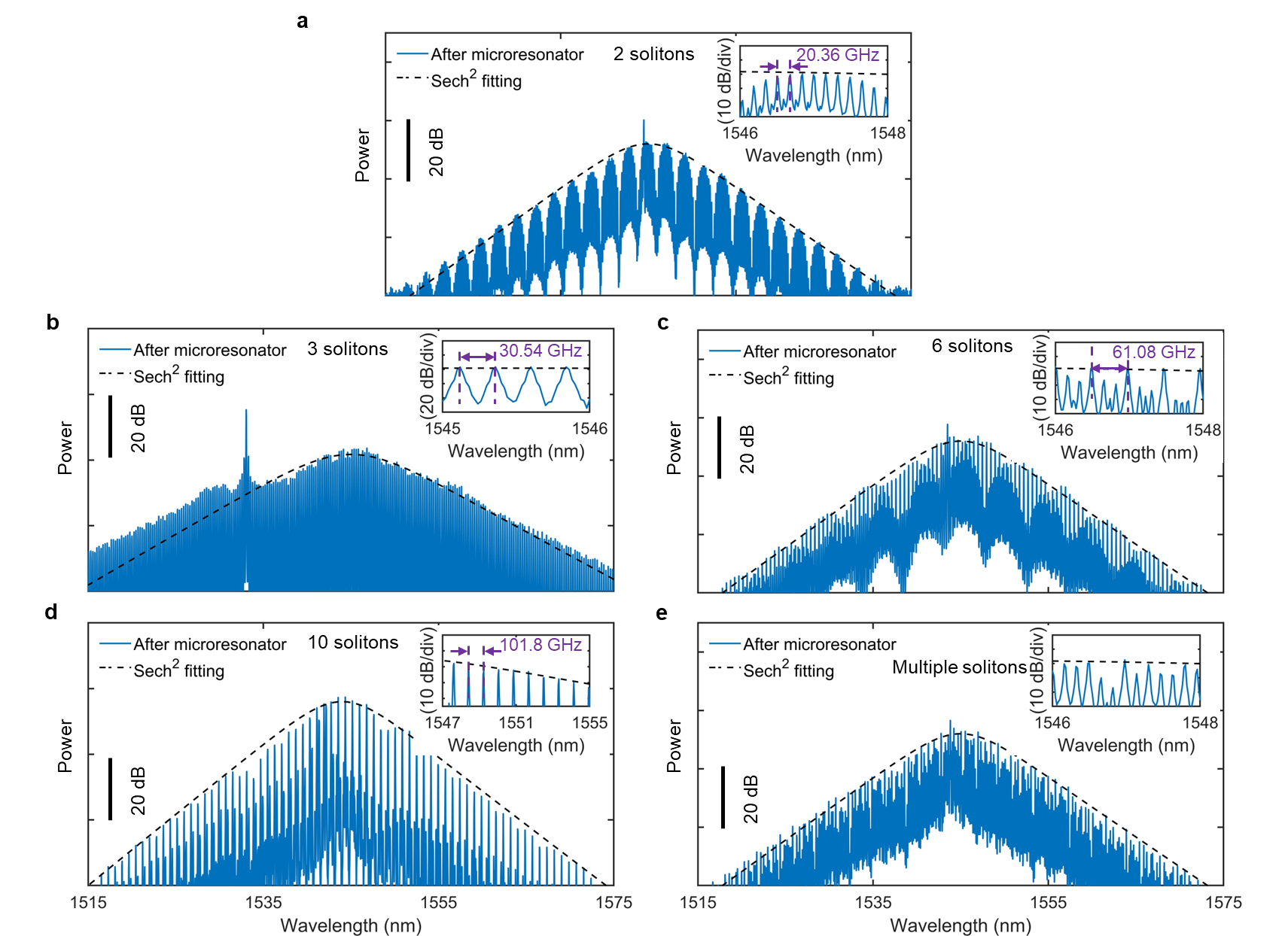


Fig. S6. Spectra for other comb states. (a) 2 solitons; (b) 3 solitons; (c) 6 solitons; (d) 10 solitons; (e) multiple solitons. Insets show the enlarged spectra.


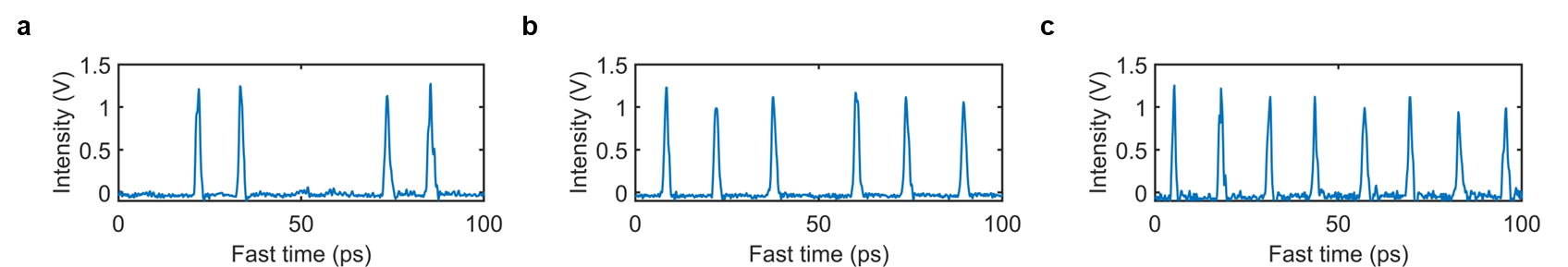


Fig. S7. Calibrated temporal traces from time magnifier system for other comb states, showing (a) 4 solitons with unequal temporal distance, (b) 6 solitons with unequal temporal distance and (c) 8 solitons with equal temporal distance.

**S8: Time magnifier setup**

The experimental setup of the time magnifier is shown in Fig. S8a. The core of the time magnifier is a parametric time lens implemented through four-wave mixing (FWM) process pumped by linearly chirped pulses. To generate the chirped pump, a mode-locked fiber laser (MLFL, Menlo C-Fiber) with a repetition rate of 50 MHz is first bandpass filtered from 1554 nm to 1563 nm (BPF2), and then chirped by a spool of dispersion compensating fiber (DCF2). The chirped pulse is then amplified by a C-band Erbium doped fiber amplifier (EDFA) to around 200 mW for pumping the time lens. On the signal input path (upper branch), the signal under test (SUT) is first bandpass filtered from 1538 nm to 1547 nm by BPF1 to limit the bandwidth. The filtered SUT is then chirped by DCF1, and then combined with the chirped pump through a wavelength division multiplexer (WDM). An example optical spectrum after the WDM is shown in the black trace of Fig. S8b, where the frequency comb is used as SUT. The combined lights are launched together into a spool of 50-m highly nonlinear fiber (HNLF, OFS), which has a zero-dispersion wavelength at 1554 nm and a nonlinear coefficient of around 12 W^-1^·km^-1^. Through FWM process inside the HNLF, an idler wave centered around 1580 nm is generated as shown in the red trace in Fig. S8b, together with other spectral components generated through other parasitic FWM processes. The polarizations of both SUT and chirped pump are controlled individually using two polarization controllers (PCs) to maximize the FWM efficiency. Since only the idler wave at 1580 nm could be regarded as the result of SUT being modulated by the time lens, it is isolated using a free-space BPF (BPF3) with more than 60-dB extinction ratio. The resulting clean idler spectrum after BPF3 is shown in the blue trace of Fig. S8b. Then, the filtered idler propagates through another spool of DCF (DCF3) to become the temporally magnified signal. This signal is pre-amplified by an L-band EDFA (EDFA2) before being detected by a fast photodetector (Newfocus 1474, 38 GHz) and a real-time oscilloscope (Tektronix DPO72504D) with 25 GHz bandwidth and 100 Gs·s^-1^ sampling rate.


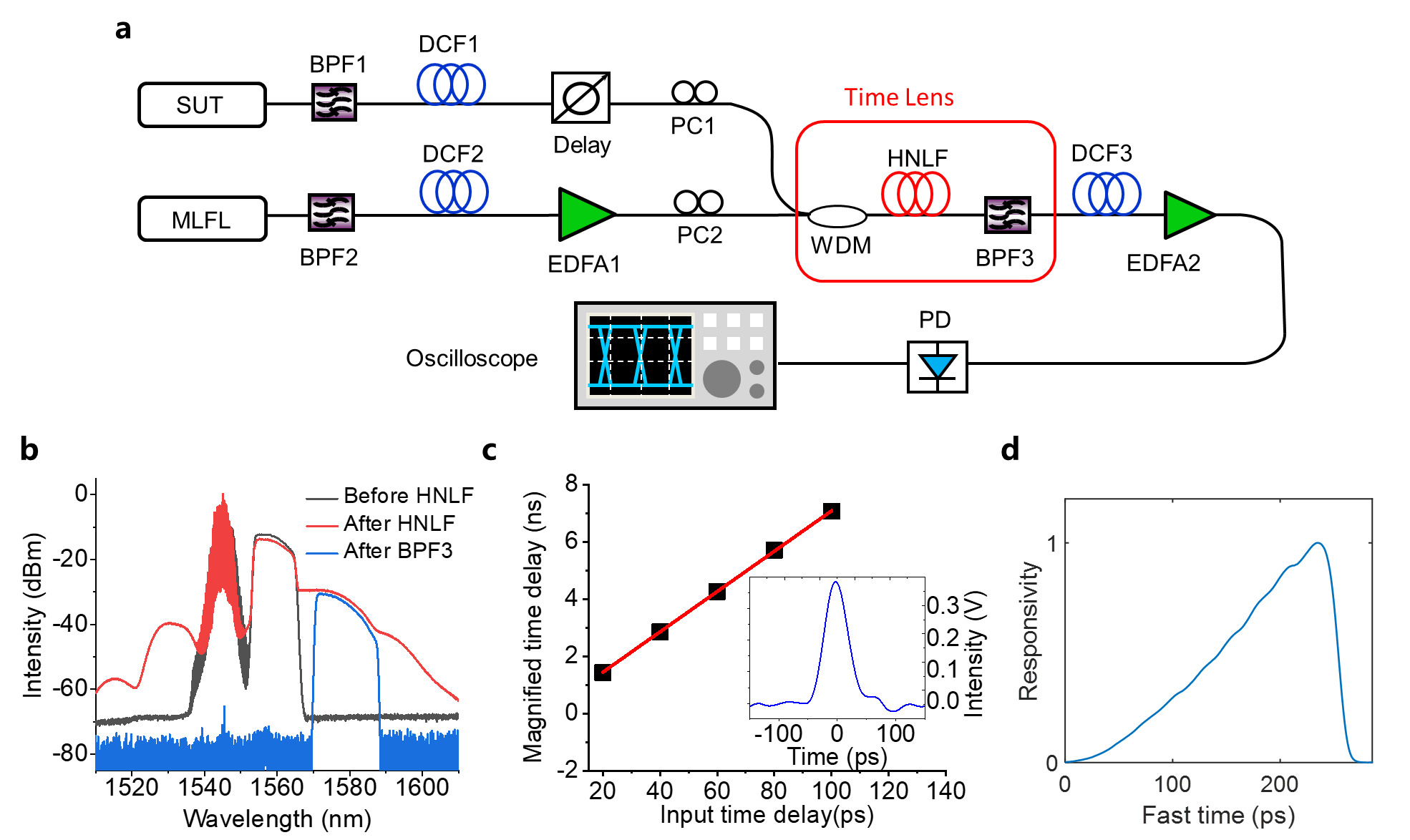


Fig. S8. (a) Experimental setup of the time magnifier. SUT, signal under test; DCF, dispersion compensating fiber; MLFL, mode-locked fiber laser; BPF, bandpass filter; EDFA, erbium-doped fiber amplifier; PC, polarization controller; WDM, wavelength division multiplexer; HNLF, highly nonlinear fiber; PD, photodetector. (b) Optical spectra related to time lens. (c) Characterization of time magnifier. (d) Responsivity of the time magnifier system.

Overall, the system functions as a temporal analog of spatial single-lens imaging system. The three spools of DCF (DCF1, DCF2, DCF3) provide input group delay dispersion (GDD) of 18.7 ps·nm^-1^ (GDD1), focal GDD of 18.9 ps·nm^-1^ (GDD2) and output GDD of 1315 ps·nm^-1^ (GDD3), respectively. Therefore, a temporal imaging condition is satisfied as

$-\frac{1}{GDD1}+\frac{1}{GDD2}=\frac{1}{GDD3}$ (S7)

and the temporal magnification ratio is determined as GDD3/GDD1 to be around 70. All the DCFs are from Corning model DCM-D-080-04, which have a third order dispersion (TOD) that is 12 times smaller than conventional single-mode fiber (e.g. SMF28), which minimize the temporal imaging aberrations.

The single-shot recording time window of our time magnifier system is essentially determined by the pump pulse width, which depends on both the pump GDD and the pump seed bandwidth. With a pump GDD of 37.8 ps·nm^-1^ (twice the focal GDD) and a pump bandwidth of 9 nm, the chirped pump pulse is calculated as 37.8 ps·nm^-1^ ×9 nm=340.2 ps. The responsivity with this time range is non-uniform due to the non-flat pump intensity cause by the gain dispersion of the EDFA and due to the non-constant four-wave mixing conversion efficiency, which narrows the single-shot recording time window of the final system to around 285 ps.

To experimentally characterize the magnification ratio and resolution of the time magnifier, the same MLFL is used as the source for both SUT and time-lens pump, which guarantees the synchronization. The relative time delay between the SUT and the pump is swept across 80 ps with a step of 20 ps using a tunable optical delay line. The corresponding magnified time delay of the output pulse is shown in Fig. S8c. A linear fitting revealed a magnification ratio of 70.4, which matched well with the design. The output pulse shape is shown in the inset of Fig. S8c, which represents a 3-dB pulse width of 48 ps. Therefore, the de-magnified output pulse width is around 680 fs. Considering the transform limited pulse width of SUT is around 390 fs at 9 nm bandwidth, the impulse response, i.e. the temporal resolution of the system can be estimated through deconvolution to be $\sqrt{680\text{f}s^{2}-390\text{fs}^{2}}=560\text{fs}$.

In the experiment, we roughly tune the synchronization between the soliton signal and the pump by observing the envelope flatness of the time magnifier output. When the soliton repetition rate is *N* *f*rep_pump+*δf*_rep_, where *f*rep_pump is the pump repetition rate and *δf*_rep_ is the repetition rate mismatch between pump and soliton, the envelope of time magnifier output shows a slow modulation at the frequency of *δf*_rep_. When we tune the pump repetition rate to make the envelope roughly flat across the total record length *T*_rec_ (100 μs under 100 Gs·s^-1^ sampling rate), it indicates that *δf*_rep_ is smaller than 1/*T*_rec_. Even though synchronization can be only achieved with a precision of *δf*_rep_ < 10 kHz using our rough tuning approach, it is sufficient to correctly unveil the soliton formation and interaction dynamics. More precise synchronization can be achieved by directly measuring *δf*_rep_ through detecting the electrical mixed signal between the frequency-divided soliton repetition rate and the pump repetition rate, which requires broadband RF frequency divider and broadband photodetector.

Due to the wavelength-dependent four-wave-mixing process (see the blue trace in Fig. S8b), the responsivity of the time magnifier system is non-uniform inside the recording time window, as shown in Fig. S8d.

**S9: Phase shift at edges of bandpass filter**

The transmission of the BPF with an ASE source shows no transmission spikes (Fig. S9).


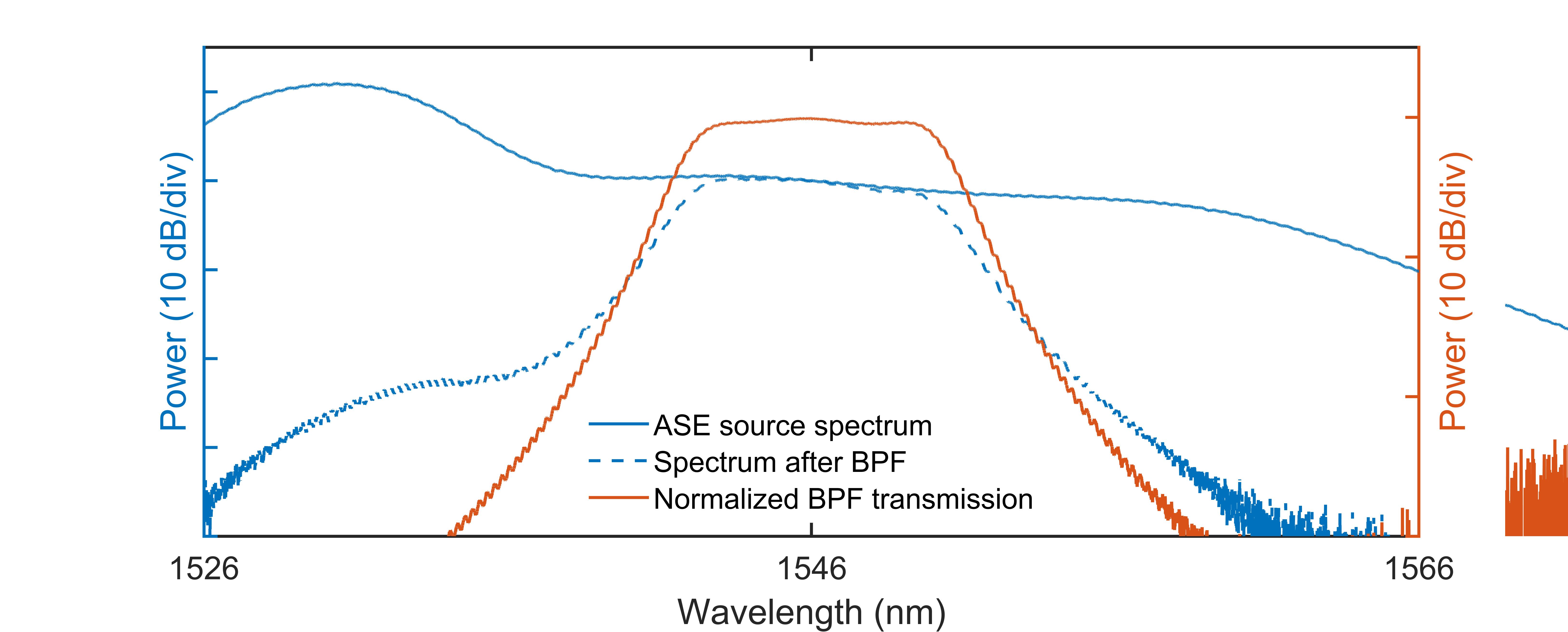


Fig. S9. Transmission of the BPF with an ASE source.

Our thin-film bandpass filter (BPF, Semrock, NIR01-1570/3-25) is a multi-cavity Fabry-Perot filter, which is a minimum phase filter with correlated amplitude and phase response through a Hilbert transform (similar to the Kramers-Krönig relations in optics) [6]. The wavelength-dependent amplitude transmission and phase response cannot be designed independently, leading to chromatic group delay. Usually, there exists a group delay for the bandpass filter edges compared to the filter center [7]. More references [8,9] shows the similar group delay. Therefore, inserted in the fiber laser cavity, there should be a phase shift between the filter center and filter edges. The two lasing single frequencies near both the short-wavelength and long-wavelength edges in Fig. 4a in the main text can verify the existence of the phase shift. The different intensities for the two lasing modes are attributed to the wavelength-dependent gain. When soliton is formed, these comb lines located at the BPF center is red detuned, while the standing out comb line is blue detuned due to the phase shift, leading to XPM comb lines.

**S10: Simulated XPM induced combs**

Figure S10 shows the simulated XPM induced combs for perfect soliton crystal with 4 solitons (a) and (b) 6 solitons (b) in Figs. 3b and 3c in the main text. The 2-soliton case is shown in Fig. 4g in the main text. Only single CW peak is considered in the simulation by coupling two LLE-like equation [Eq. (S5)]: one governing the main soliton combs, the other governing the XPM combs. The small discrepancy of the XPM comb bandwidth between the experimental results and simulation results is attributed to the neglecting the wavelength-dependent phase shift of the bandpass filter.


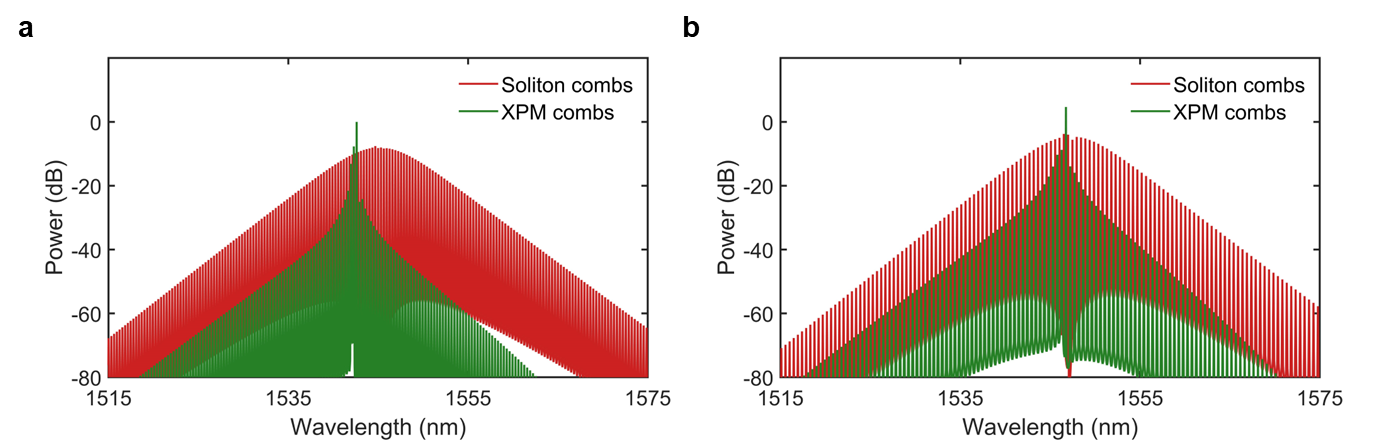


Fig. S10. Simulated XPM induced combs for perfect soliton crystal with 4 solitons (a) and (b) 6 solitons (b).

**S11: Long-term stability of the soliton microcombs**

The long-term stability of the soliton microcombs is determined by the stable detuning, which is the relative position between the lasing frequencies in the large fiber cavity and the microresonator resonance. Therefore, it is important to control the microresonator temperature and the optical path length in the large fiber cavity, which are determined by both the external temperature-controlled oven and the intracavity power. Of note, the optical path length can change due to the intracavity power fluctuation through gain saturation effect [10]. In order to achieve high long-term stability, it is better to enclose and temperature-control both the EDFA and the microresonator. In addition, low relative intensity noise (RIN) 980-nm pump laser should be used in EDFA. Besides the aforementioned passive methods, similar to the conventional Kerr soliton microcombs, active feedback loops can be introduced to improve the long-term stability by modulating either the pump power of the EDFA, the intracavity power or the delay line. With these passive and active methods implemented, we believe the long-term stability of soliton microcombs in the microresonator-filtered fiber lasers is comparable to that for conventional Kerr soliton microcombs.

In our experimental setup, the home-built EDFA is enclosed in a box made of acrylic sheet and is largely isolated from the lab environment, including the phonons and heat change through the air. The EDFA is not temperature controlled, while the microresonator is temperature controlled with a resolution of 10 mK. The long-term temperature variation in the lab is ±1 °C. As we describe in the main text, the soliton microcombs can keep stable for tens of minutes with repetition rate fluctuations in a range of 20 kHz for the 2-FSR microcombs.

**S12: Temporal evolution for state IV**

As shown in Fig. S12. we plot the temporal evolution from the time magnifier system for state IV in Fig. 4a in the main text. Noisy pulses can be formed, which is a precursor for mode locking. However, the pulses can not keep stable since they are in the unstable MI regime. Of note, the unstable soliton can last for multiple roundtrips of the large fiber laser cavity, which can be attributed to the long photon decay time in the microresonator and small nonlinearity in the EDFA.


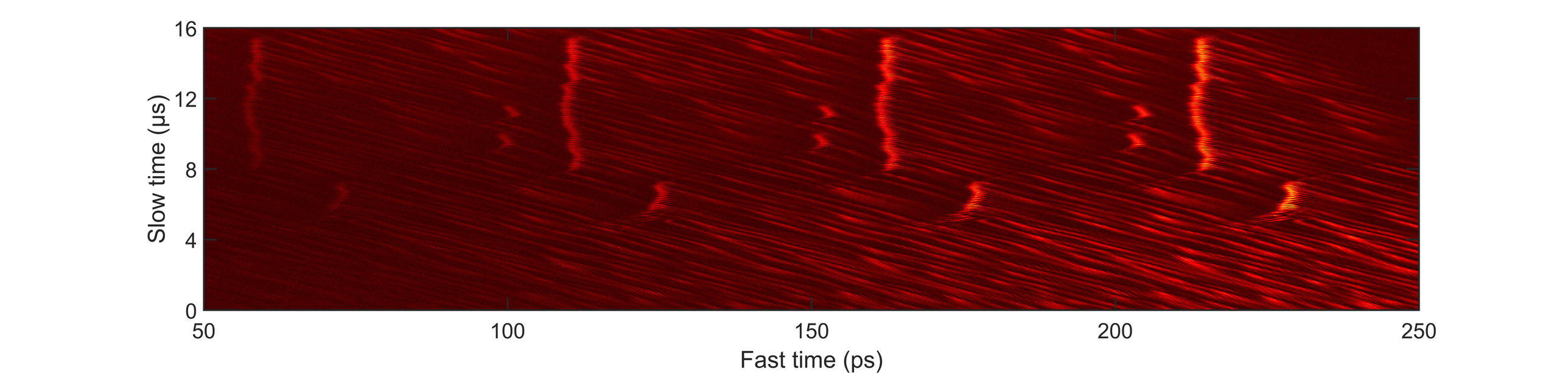


Fig. S11. Temporal evolution from the time magnifier system for state IV in Fig. 4a in the main text.

**S13: Linewidth measurement of the FFP microresonator**

The transmission and reflection spectrum of the FFP microresonator is calibrated by an unbalanced Mach–Zehnder interferometer (MZI) with a free spectral range (FSR) of 1 MHz, as shown in Fig. S12.


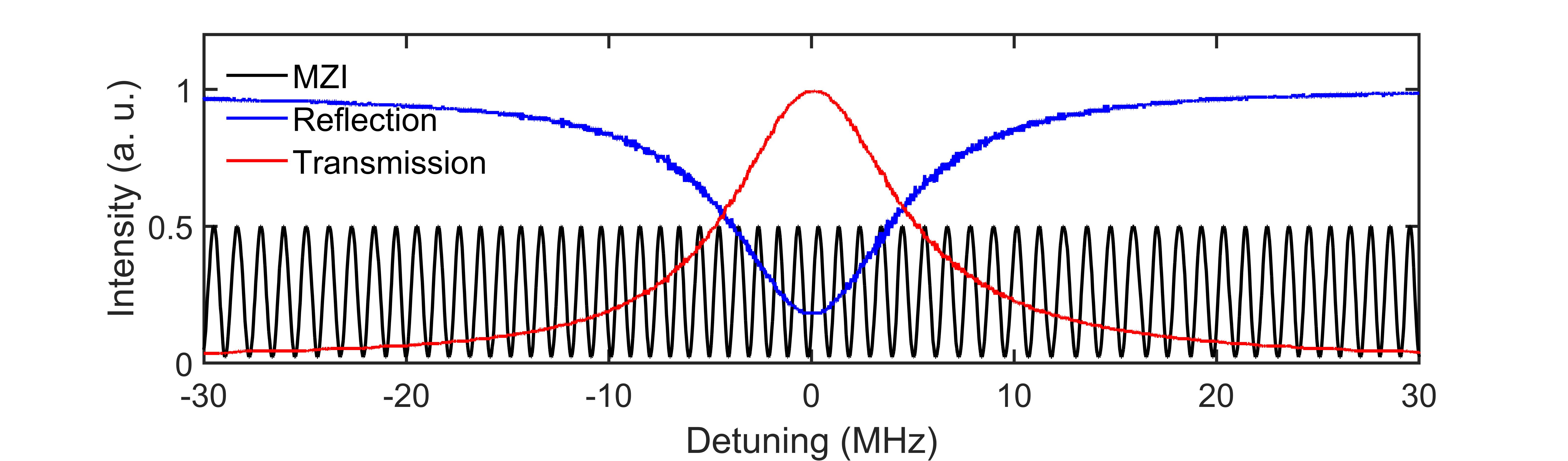


Fig. S12. Transmission and reflection spectrum calibrated by MZI.

**References**

[1] X. Xue, M. Qi, A.M. Weiner, Normal-dispersion microresonator Kerr frequency combs, Nanophotonics. 5 (2016) 244–262. https://doi.org/10.1515/nanoph-2016-0016.

[2] M. Anderson, F. Leo, S. Coen, M. Erkintalo, S.G. Murdoch, Observations of spatiotemporal instabilities of temporal cavity solitons, Optica. 3 (2016) 1071–1074. https://doi.org/10.1364/OPTICA.3.001071.

[3] X. Xue, Y. Xuan, Y. Liu, P.-H. Wang, S. Chen, J. Wang, D.E. Leaird, M. Qi, A.M. Weiner, Mode-locked dark pulse Kerr combs in normal-dispersion microresonators, Nat. Photonics. 9 (2015) 594–600.

[4] A. Pasquazi, M. Rowley, P.-H. Hanzard, A. Cutrona, H. Bao, J.S.T. Gongora, M. Peccianti, S. Chu, B. Little, D. Moss, others, Self-Emergence of Robust Micro Cavity-Solitons, (2022). https://doi.org/10.21203/rs.3.rs-468461/v1.

[5] Y.O. Barmenkov, A.V. Kir’yanov, M.V. Andrés, Resonant and thermal changes of refractive index in a heavily doped erbium fiber pumped at wavelength 980 nm, Appl. Phys. Lett. 85 (2004) 2466–2468. https://doi.org/10.1063/1.1787151.

[6] M. Gerken, D.A. Miller, Multilayer thin-film coatings for optical communication systems, in: Opt. Interf. Coat., Optica Publishing Group, 2004: p. ThD2. https://doi.org/10.1364/OIC.2004.ThD2.

[7] K. Zhang, J. Wang, E. Schwendeman, D. Dawson-Elli, R. Faber, R. Sharps, Group delay and chromatic dispersion of thin-film-based, narrow bandpass filters used in dense wavelength-division-multiplexed systems, Appl. Opt. 41 (2002) 3172–3175. https://doi.org/10.1364/AO.41.003172.

[8] R.B. Sargent, Recent advances in thin film filters, in: Opt. Fiber Commun. Conf., Optica Publishing Group, 2004: p. TuD6.

[9] X. Cui, X. Zheng, Y. Li, Y. Xu, S. Liu, Research on phase reconstruction and chromatic dispersion characteristic of thin film filters, Opt. Commun. 256 (2005) 123–131. https://doi.org/10.1016/j.optcom.2005.06.069.

[10] M. Janos, S.C. Guy, Signal-induced refractive index changes in erbium-doped fiber amplifiers, J. Light. Technol. 16 (1998) 542. https://doi.org/10.1109/50.664061.
